# Single transitions and persistence of unemployment are associated with poor health outcomes

**DOI:** 10.1186/s12889-019-7059-8

**Published:** 2019-06-13

**Authors:** Gerrie-Cor Herber, Annemarie Ruijsbroek, Marc Koopmanschap, Karin Proper, Fons van der Lucht, Hendriek Boshuizen, Johan Polder, Ellen Uiters

**Affiliations:** 1 Centre for Nutrition, Prevention and Health Services, National Institute of Public Health and the Environment, PO Box 1, 3720 BA Bilthoven, The Netherlands; 2 Centre for Health and Society, National Institute of Public Health and the Environment, Bilthoven, The Netherlands; 3 Erasmus School of Health Policy and Management, Erasmus University Rotterdam, Rotterdam, The Netherlands; 4 Division of Human Nutrition, Department of Agrotechnology and Food Sciences, Wageningen University, Wageningen, The Netherlands; 5 Tilburg School of Social and Behavioral Sciences, Tilburg University, Tranzo, Tilburg, The Netherlands

**Keywords:** Unemployment, Transitions, Health, Health related behavior, Economic recession, Longitudinal study

## Abstract

**Background:**

Although job loss has been associated with decline in health, the effect of long term unemployment is less clear and under-researched. Furthermore, the impact of an economic recession on this relationship is unclear. We investigated the associations of single transitions and persistence of unemployment with health. We subsequently examined whether these associations are affected by the latest recession, which began in 2008.

**Methods:**

In total, 57,911 participants from the Dutch Health Interview Survey who belonged to the labour force between 2004 and 2014 were included. Based on longitudinal tax registration data, single employment transitions between time point 1 (t1) and time point 2 (t2) and persistent unemployment (i.e. number of years individuals were unemployed) between t1 and time point 5 (t5) were defined. General and mental health, smoking and obesity were assessed at respectively time point 3 (t3) and time point 6 (t6). Logistic regression models were performed and interactions with recession indicators (year, annual gross domestic product estimates and regional unemployment rates) were tested.

**Results:**

Compared with individuals who stayed employed at t1 and t2, the likelihood of poor mental health at the subsequent year was significantly higher in those who became unemployed at t2. Persistent unemployment was associated with poor mental health, especially for those who were persistently unemployed for 5 years. Similar patterns, although less pronounced for smoking, were found for general health and obesity. Indicators of the economic recession did not modify these associations.

**Conclusions:**

Single transitions into unemployment and persistent unemployment are associated with poor mental and general health, obesity, and to a lesser extend smoking. Our study suggests that re-employment might be an important strategy to improve health of unemployed individuals. The relatively extensive Dutch social security system may explain that the economic recession did not modify these associations.

## Background

The relevance of health for economic and community participation has been confirmed by many scholars [1, 2]. The phrase health is wealth, and wealth is health clearly summarises the reciprocal character of this relationship. With respect to economic participation, evidence suggests that employment is beneficial for health [3–7], in particular for depression and mental health [8, 9]. Unemployment, on the other hand, has been linked to poorer self-rated health [10–12], mental illness and depression [8, 10, 12], as well as increased morbidity and mortality [10]. Although the underlying mechanisms are unclear, three possible explanations are mentioned in the literature for the relation between becoming unemployed involuntary and poorer health. Given the consistent association between wealth and health, a decline of income (sometimes into poverty) because of becoming unemployed is one explanation. Another explanation is offered by stress induced by unemployment e.g. by loss of self-esteem and a time structure to their days. A third explanation stems from the possibility that unemployed individuals have been found to show more self-destructive behaviour, ranging from increased levels of smoking and drinking to self-destructive behaviour like (attempted) suicide [4, 13, 14].

Furthermore, although some studies suggest the relationship between job loss and health and health behaviour to be causal, this issue is still debated [3, 15–17]. For instance, while some evidence exists for that unemployment is causally related to an increase in smoking, this association is poorly understood, which also applies for other health behaviours, such as obesity [18]. There is also support that the association between unemployment and health reflects a selection effect, which is implying that individuals with poorer health are more likely to become and stay unemployed [19–21]. The latter is underlined by findings of studies differentiating between the reasons for entry into involuntary unemployment. Some studies report no negative health effects after job loss due to exogenous reasons (such as the closure of business), which suggests that a selection effect is likely to explain the association between unemployment and health [22, 23].

Studying the relation between employment and health is further complicated by the fact that, because of the increase in flexibility at the labour market, people can experience multiple unemployment spells during their working life, leading to long-term unemployment. Although a link between persistence in unemployment and poorer health seems plausible [24–26], these studies are limited by the relatively small sample size and the use of self-reported data on exposures and outcomes. Furthermore, it is unclear how the persistence of unemployment over time might influence health outcomes [24]. Three different possibilities of development have been proposed in the literature. The first model relates to a dose-response relation, where increasing persistence of unemployment is followed by a further deterioration in health. The second model suggest the development of a steady-state situation- i.e. after a certain level of effect additional unemployment will not add anything more. The final model suggests immunity to repeated exposure of unemployment – i.e. after sometime a maximum is reached and that any more unemployment will result in lower effect estimates [24].

It has also been suggested that the health impact of unemployment may depend on macro-level economic circumstances, such as an economic recession [27–30]. On the one hand, involuntary job loss during an economic recession may be less detrimental to health, because unemployment is less rare and the stigma of unemployment that could potentially harm (mental) health decreases [31]. On the other hand, the impact of unemployment on health may be increased during an economic recession as it may lead to increased insecurities about the chances for reemployment and therefore to increased psychological distress [32]. Previous studies have suggested that the post-2008 economic recession intensified the negative impact of unemployment on general and mental health [31, 33, 34] and that differences with respect to unhealthy behaviour, such as smoking and drinking, increased between the employed and unemployed [34]. The existing evidence regarding the relationship between the economic recession and health is most consistently found for suicides and mental health [27]. However, until now, few studies have investigated whether the associations between the degree in persistence of unemployment and health (behaviour) differed during and after the economic recession that started in 2008. E.g. one study reported that the decline in health is in particular steep for people who have been unemployed for several years [35], while another study found job-loss and long-term employment to be risk factors for substance use in times of economic recession [28].

This paper aims to provide more insight in the complex relationship between employment status and health, without trying to make causal statements. In this observational study, we examine the associations between single transitions in employment status and persistence of unemployment and health and health-related behaviour. Subsequently, we examine whether the associations between single transitions in employment status and persistence of unemployment and health (behaviour) are affected by the latest recession, which began in 2008.

## Methods

### Study population

Annual data on employment status and personal gross income were obtained from tax registers from Statistics Netherlands between 2004 and 2015 (more details below). Data on health status and behaviour were obtained from the Dutch Health Interview Survey (HIS), collected between 2005 and 2015 by Statistics Netherlands. The HIS is a repeated cross-sectional survey of the developments in health, medical contacts, lifestyle and preventive behaviour of the Dutch population. Throughout the year, a random sample of the Dutch population, aged 0 years or older and living in non-institutionalised households, is drawn from the Dutch population register (approximately 15,000 persons/year). The survey consists of two parts. The first part includes questions on background variables such as sex, age, perceived general health, height, weight, and smoking. The second part includes questions on mental health. At the end of the first part, respondents can decide whether or not to participate in the second part of the survey. The annual response rate is 60–65%; around 55–80% of these respondents also respond to the additional questionnaire. In the present analyses, the study population consisted of adults who were registered in the tax data between 2004 and 2014 and responded to the HIS surveys between 2005 and 2015 (*n* = 76.629).

### Health and health-related behaviour

The outcomes of interest were general health, mental health, smoking and obesity. Based on answers to the question: “In general how is your health”, general health was categorised as good (good or very good) or less than good (acceptable, bad or very bad). Mental health was determined with the Mental Health Inventory (MHI-5) [36]. Answers ranged from ‘all the time’ to ‘never’ on a six-point scale [36]. The MHI-5 was dichotomized into good (≥ 60) and less than good (< 60) [37]. Smoking was asked with a single question (Do you sometimes smoke?) and divided into smokers (yes) and non-smokers /ex-smokers (no) to indicate current smokers. Body Mass Index (BMI) was computed as self-reported weight (kg)/height (m^2^). Obesity, defined as BMI > = 30 kg/m^2^ was used as dependent variable in our analyses.

### Transitions and persistence in employment status

**Fig. 1:**
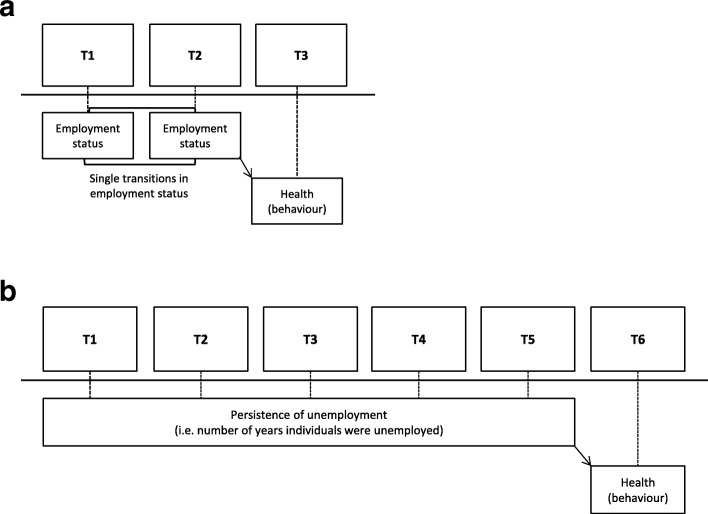
Visualizing the logistic regression models with time lag to study associations between **a**) single transitions in employment status and health (behaviour) and **b**) persistence of unemployment and health (behaviour)

Current employment status was categorized as employed (those whose main annual resource of income was from labour, as either an employee or an entrepreneur) and unemployed (those whose main annual resource of income was from social benefits). Students, pension recipients, disabled people and individuals who had no income were excluded. Single transitions in employment status between time point 1 (t1) and time point 2 (t2) were then constructed and categorised into four categories: staying employed; becoming employed; becoming unemployed or staying unemployed. In addition, a variable was constructed for degree of persistence in unemployment (i.e. the number of years individuals were unemployed) between t1 and time point 5 (t5). This score ranged from 0 (persistently employed) to 5 (persistently unemployed). For the analysis with single transitions in employment status as the exposure, we included those who had data on employment status at t1 and time point 2 (t2) and who had health measured at the subsequent year (time point 3; t3), giving a total of 57,904 (general health), 40.401 (mental health), 57,911 (obesity) and 57,906 (smoking) persons (Fig. 1a). Similarly, for the analysis with persistence of unemployment as the exposure, we included those who had data on employment status between t1 and t5 and who had health measured at the subsequent year (time point 6; t6), giving a total of 39,168 (general health), 26,368 (mental health), 39,174 (obesity) and 39,171 (smoking) persons (Fig. 1b).

### Other variables

The following socioeconomic variables were included in the analysis as potentially confounding variables. Education was categorized into low (lower or equal to secondary education), intermediate (higher secondary education), or high (higher professional education and university). Household position of each respondent was classified as breadwinner, partner, child or other household member. Standardised disposable personal income, based on tax registration data, indicates the purchasing power of a person.

### Economic recession

We focus on the most recent economic recession of 2008 in the Netherlands [34, 38]. To test whether the recession modified the associations between single transitions in employment status and persistence in unemployment and health, we added interaction terms with three indicators of the recession. First, a categorical variable was created to specify whether participants were in the pre-crisis (2005–2007), crisis (2008–2013) or post-crisis (2014–2015) period in the latest year of their transition in work status, i.e. t2 and t5 for single transitions and persistent unemployment respectively. The second and third indicators were annual gross domestic product (GPD) estimates and annual unemployment rates per province – i.e. 12 established areas in the Netherlands - between 2004 and 2015, respectively. The latter indicator was used because employment perspectives varies between provinces.

### Statistical analysis

Logistic regression models were performed to calculate odds ratio’s (ORs) with 95% confidence intervals (CIs) for the associations between single transitions (i.e. between t1 and t2) in employment status and persistence in unemployment (i.e. between t1 and t5) with health status and behaviour (model 1). Time lagged models were used to ensure that transitions in employment status were associated with health status and behaviour at the subsequent year (Fig. 1a and b). In both analyses, the group of respondents staying employed was set as the reference category. In model 2, we adjusted for age, sex, education and household position. To examine the possible influence of income on the association between employment transitions and health, we additionally adjusted for personal gross income (model 3). Data on age, sex, education, household position and personal gross income collected at the time of the health survey were used. To account for the last registered work status in the analysis of persistence of unemployment, we additionally adjusted for individual’s employment status (i.e. employed or unemployed) at the time of the health survey in all three models. We formally tested effect modification by the economic recession by adding interaction terms between each of the single transitions and persistence of unemployment and a categorical indicator for whether participants contributed data in the pre-crisis, crisis or post-crisis period. By way of sensitivity analyses, interaction terms with two other indicators of the economic recession were added as well, i.e. 1) annual GPD estimates and 2) annual regional unemployment rates. Finally, since odds ratios do not allow for a direct interpretation of the size of the associations, we computed the standardised prevalence of the final model (model 4) for each outcome (standardised for the co-variates). Analyses were conducted on dichotomous variables because in some of the exposure groups we did not have enough power to further differentiate the outcomes, i.e. in those who were persistently unemployed, due to low numbers. However, a study that compared dichotomized general health against general health in categorized indicated similar results in relation to socioeconomic position [39]. Furthermore, we performed a secondary analysis where we used BMI as a continuous outcome variable, and found almost identical results. These findings indicate that dichotomization in this study is likely to be justified.

## Results

Of 57,904, 40,401, 57,911 and 57,906 persons who had data on employment status (at t1 and t2) and outcomes (at t3), respectively, persons with missing data on covariates were excluded. The final sample sizes in the analyses on single transitions in employment status were *n* = 47,734 (82.4%; general health), 32,919 (81.5%; mental health), 47,739 (82.4%; obesity) and 47,734 (82.4%; smoking). Similarly, of 39,168, 26,368, 39,174 and 39,171 persons who had data on employment status (between t1 and t5) and outcomes (at t6), respectively, data from 29,866 (76.3%; general health), 19,593 (74.3%; mental health), 29,870 (76.2%; obesity) and 29,867 (76.2%; smoking) were included in the analyses on persistence of unemployment. Individuals who were excluded due to incomplete data in the analysis on single transitions in employment status and general health were older, less likely to be lower educated, but generally less healthy than those who were included in the analyses (Appendix 1). Similar differences were observed for the other outcomes (results not shown).

In 2005, 52% of our study population were men with a mean age of 42.5 years. Compared with individuals who remained employed between 2004 and 2005, those who found a job, became unemployed or remained unemployed between 2004 and 2005 were less educated, more likely to be a current smoker and to have a poor perceived and mental health at the subsequent year. Those who remained unemployed between 2004 and 2005 were more likely to be female and obese than those who stayed employed and to be the least healthy, with almost 50% reporting being in poor general health and 43% reporting poor mental health (Table 1).

**Table 1:** Characteristics of the total population in 2005 according to single transitions in employment status between 2004 and 2005

		Transitions in employment status between 2004 and 2005			
	Total population (*N* = 57,911)	Employed-employed (*N* = 4260)	Unemployed-employed (*N* = 76)	Employed-unemployed (*N* = 73)	Unemployed-unemployed (*N* = 264)
Age, y (mean ± SD)	42.5 ± 12.3	40.5 ± 12.0	35.5 ± 9.7	43.8 ± 11.7	44.3 ± 13.0
Men (%, N)	52.4 (30322)	55.5 (2363)	46.1 (35)	65.8 (48)	40.9 (108)
Low education (%,N)	8.8 (4564)	39.5 (1680)	35.5 (27)	28.8 (21)	20.1 (53)
BMI ≥ 30 k/m^2^ (%,N)	10.5 (6085)	9.3 (395)	7.9 (6)	11.0 (8)	13.6 (36)
Poor perceived health (%,N)	15.6 (9024)	13.4 (570)	26.3 (20)	20.6 (15)	49.6 (131)
Poor mental health (%,N)	11.4 (4619)	10.3 (367)	28.6 (14)	19.6 (10)	43.3 (78)
Current smokers (%,N)	29.4 (17018)	32.7 (1391)	48.7 (37)	45.2 (33)	48.1 (127)

### Transitions in employment status and mental health

In the crude model, compared with individuals who stayed employed, odds of poor mental health at the subsequent year were significantly higher in those who found a job (Odds Ratio (OR) 2.50, 95% confidence interval (CI) 1.96, 3.18), became unemployed (OR 3.10, 95% CI 2.53, 3.79), or stayed unemployed (OR 6.17, 95% CI 5.55, 6.84; P_trend_ < 0.001; Table 2). Adjustment for possible confounders in model 2 attenuated these associations, but all associations with mental health remained significant (Table 2), even after adjustment for personal gross income. As compared to those who stayed employed, the ORs were 2.01 (95% CI 1.52, 2.66), 3.08 (95% CI 2.45, 3.86) and 4.53 (95% CI 4.00, 5.15) in those who found a job, became unemployed or stayed unemployed, respectively (P_trend_ < 0.001; Table 2).

**Table 2:** Odds Ratios (95% confidence intervals) for the association between single transitions in employment status and persistence of unemployment and poor mental health

	Model 1 (crude model)	Model 2 (age, sex, education, household position)	Model 3 (+ personal gross income)
Single transitions in employment status^a^			
Employed-employed	1.00 (Reference)	1.00 (Reference)	1.00 (Reference)
Unemployed-employed	2.50 (1.96,3.18)*	2.08 (1.58,2.75)*	2.01 (1.52,2.66)*
Employed-unemployed	3.10 (2.53,3.79)*	3.15 (2.51,3.95)*	3.08 (2.45,3.86)*
Unemployed-unemployed	6.17 (5.55,6.84)*	5.04 (4.45,5.71)*	4.53 (4.00,5.15)*
P-trend	< 0.001	< 0.001	< 0.001
Persistence of unemployment^bc^			
0 (=persistently employed)	1.00 (Reference)	1.00 (Reference)	1.00 (Reference)
1	1.68 (1.30,2.17)*	1.42 (1.08,1.87)*	1.38 (1.05,1.81)*
2	2.56 (1.85,3.56)*	2.38 (1.69,3.35)*	2.25 (1.60,3.17)*
3	3.30 (2.23,4.90)*	2.83 (1.86,4.31)*	2.55 (1.67,3.89)*
4	3.10 (1.87,5.13)*	2.41 (1.42,4.11)*	2.14 (1.26,3.65)*
5 (=persistently unemployed)	8.40 (5.90,11.95)*	5.60 (3.83,8.17)*	4.68 (3.19,6.86)*
P-trend	< 0.001	< 0.001	< 0.001

^a^Single transitions in employment status relate to the work transitions in the previous 2 years. ^b^ Persistence in unemployment relates to the number of years individuals were unemployed in the previous 5 years. ^c^ To account for the last registered work status in the analysis of persistence of unemployment, we additionally adjusted for individual’s employment status (i.e. employed or unemployed) at the time of the health survey in all three models.* *p* ≤ 0.05

Analysis of persistence of unemployment showed that in general, the odds of poor mental health became higher with increasing degree of persistence of unemployment (Table 2). As compared to those who were persistently employed, the OR for poor mental health was 1.68 (95% CI 1.30, 2.17) in those who became unemployed once and 8.40 (95% CI 5.90, 11.95) in those who were persistently unemployed for 5 years (Model 1; P_trend_:< 0.001; Table 2). Including age, sex, education and household position into the models reduced these associations, in particular among those who were persistently unemployed and the OR became 5.60 (95% CI 3.83, 8.17; P_trend_:< 0.001) in comparison with persistently employed participants. After adding personal gross income to the models, the OR further decreased to 4.68 (95% CI 3.19, 6.86; P_trend_:< 0.001; Table 2).

### Transitions into (un) employment and general health and health related behaviour

In general, similar patterns were found for poor general health, obesity and current smoking although ORs were lower for obesity and current smoking. Compared with individuals who stayed employed between t1 and t2, the OR of obesity at the subsequent year was 1.49 (95% CI 1.32, 1.68; P_trend_ < 0.001) in those who stayed unemployed (Model 2; Table 3). Additional adjustment for personal gross income hardly changed this estimate (OR 1.50, 95% CI 1.37, 1.65; P_trend_ < 0.001; Appendix 2). Compared with persistently employed (i.e. employed for 5 years) individuals, the adjusted OR of obesity was 2.03 (95% CI 1.46, 2.82) in those who were persistently unemployed in the 5 year period (Table 3). Again, further adjustment for personal gross income did not change this estimate (OR 2.13, 95% CI 1.53, 2.98; P_trend_ < 0.001; Appendix 2).

**Table 3:** Odds Ratios and 95% confidence intervals for the associations between single transitions in employment status and persistence of unemployment and poor general health, obesity and smoking^a^

	Poor general health	Obesity	Smoking
Single transitions in employment status^b^			
Employed-employed	1.00 (Reference)	1.00 (Reference)	1.00 (Reference)
Unemployed-employed	1.62 (1.30,2.01)*	1.31 (1.01,1.69)*	1.39 (1.16,1.67)*
Employed-unemployed	1.93 (1.60,2.31)*	1.08 (0.84,1.37)	1.77 (1.51,2.07)*
Unemployed-unemployed	5.14 (4.68,5.65)*	1.49 (1.32,1.68)*	1.50 (1.37,1.65)*
P-trend	< 0.001	< 0.001	< 0.001
Persistence of unemployment in the past 5 years^cd^			
0 (=persistently employed)	1.00 (Reference)	1.00 (Reference)	1.00 (Reference)
1	1.20 (0.98,1.47)	1.31 (1.05,1.64)*	1.82 (1.55,2.14)*
2	1.65 (1.27,2.13)*	1.45 (1.07,1.96)*	1.52 (1.22,1.91)*
3	2.00 (1.46,2.76)*	1.31 (0.88,1.95)	1.63 (1.21,2.18)*
4	3.28 (2.27,4.76)*	1.42 (0.88,2.30)	1.90 (1.33,2.70)*
5 (=persistently unemployed)	6.11 (4.60,8.11)*	2.03 (1.46,2.82)	1.33 (1.04,1.71)*
P-trend	< 0.001	< 0.001	0.05

^a^Adjusted sex, age, education, unemployment in the region and household position

^b^Single transitions in employment status relate to the work transitions in the previous 2 years. ^c^ Persistence in unemployment relates to the number of years individuals were unemployed in the previous 5 years. ^d^ To account for the last registered work status in the analysis of persistence of unemployment, we additionally adjusted for individual’s employment status (i.e. employed or unemployed) at the time of the health survey

*p ≤ 0.05

For current smoking, the pattern was less clear, as the adjusted OR of current smoking was 1.90 (95% CI 1.33, 2.70) in those who were unemployed for 4 years and 1.33 (95% CI 1.04, 1.71) in those who were persistently unemployed (Model 2; P_trend_ 0.05; Table 3). Additional adjustment for personal gross income attenuated these findings and ORs were significantly increased for those who were unemployed for up to 4 years (OR 1.76, 95% CI 1.24,2.52), but not thereafter (OR 1.20, 95% CI 0.93,1.54; P_trend_ 0.25; Appendix 2).

### Interactions with recession indicators

Formal tests for interaction between a categorical recession indicator (i.e. before, during or after the 2008–2013 recession), single transitions and persistence in unemployment and mental health, general health, obesity and smoking did not provide sufficient evidence for stratified analyses (P for interactions > 0.15). In addition, sensitivity analysis in which interactions with two other indicators of the post-2008 economic recession (i.e. annual GPD estimates and annual regional unemployment rates) were tested generally corroborated with these findings (results not shown).

### Standardised prevalence

Table 4 shows the standardised prevalence for poor mental health, poor general health, obesity and smoking. If the whole study population stays employed in 2 years’ time (employed-employed), 10% would report having poor mental health and 13% would report having poor general health. The prevalence of poor mental and general health increases to 33 and 41% respectively if the whole study population stays unemployed (unemployed-unemployed).

**Table 4:** Standardised prevalence of poor mental health, poor general health, obesity and smoking for each single transition in employment status

	Poor mental health	Poor general health	Obesity	Smoking
Single transitions in employment status				
Employed-employed	0.10	0.13	0.10	0.29
Unemployed-employed	0.18	0.19	0.13	0.36
Employed-unemployed	0.25	0.22	0.11	0.42
Unemployed-unemployed	0.33	0.41	0.15	0.38

For obesity, the pattern is less steep with 10% reporting being obese if everyone stayed employed, up to 15% being obese with all participants being unemployed. Finally, if the whole study population stays employed, almost 29% of the population would smoke. If the whole population makes the transition from unemployed into employed, or from employed into unemployed or stays unemployed, between 38 and 42% of the whole population would smoke.

## Discussion

In this observational study, we found that employment transitions are associated with individuals’ health and health related behaviour. Compared with individuals who were persistently employed, individuals who lost their job and especially those who were persistently unemployed for up to 5 years were more likely to report poor mental and general health. The associations with obesity and smoking followed the same pattern, although less pronounced, especially for smoking. These associations seemed to be partly, but not fully attributable to a range of confounders and personal income.

Our results on single transitions showed that the unemployed who transited to work scored significantly more favourable on general and mental health as well as on smoking and obesity than those who either became or remained unemployed. Furthermore, the likelihood of poor mental and general health and obesity became higher with increasing degree of persistence of unemployment in the previous 5 years, indicating that chains of job loss-events are important for health as well. This is in line with previous studies indicating that individuals who are more persistently unemployed are in poorer health than the stable employed [24–26, 40]. Odds of smoking, however, became higher up until 4 years of unemployment, but no longer thereafter. Although these findings are possibly explained by the development of a steady-state situation after years of unemployment [24], our observational data do not allow for definitive conclusions.

Our study dealt with the association between health and both exit from and entry into employment simultaneously; a similar approach was used in a few studies so far [21, 40, 41]. Our findings provide additional evidence for that gaining or regaining employment is beneficial for health. Hence, reemployment might be an important strategy to improve the health of unemployed individuals. This is in line with previous research [4, 8]. Due to our study design, however, we were unable to identify causative relationships between unemployment and health. Therefore, the directions of the associations remain unclear. For instance, it is not clear if unemployment is associated with poor health or if good health is a prerequisite for getting and keeping a job. For both directions, support is available in the literature [19]. Further research is required to study the possible influence of this ‘healthy worker effect’ on the observed associations.

Our finding that the associations between single transitions and persistence in unemployment and poor health are partly accounted for by income is in keeping with findings of a recent longitudinal study showing that income is not a mediator of the relationship between unemployment and health [29]. Further research is required to study whether other intermediates, such as financial strain, as suggested by Tøge [29] may explain the associations.

Our finding that the associations between work transitions and health did not vary by times of an economic recession is novel. This is not in keeping with results of several previous studies conducted in Europe and North America, which reported an intensification of the health damaging effects of job loss during economic recessions [31, 33, 34, 38, 42, 43]. Other studies conducted in Finland and Sweden, however, concluded that in times of an economic recession the negative health impact of work transitions was less severe [44, 45] or absent [46]. Prior evidence suggests that possible detrimental effects of economic recessions can be mitigated by social welfare programs [47–50]. Hence, it is possible that social benefits play a protective role for health during economic changes. Perhaps the Dutch social security system, which is relatively extensive compared to other countries, buffered the negative effects of the economic recession on the association between unemployment and health and related behaviour. Another explanation for the non-existent impact of the recession in our study is that the recession may change the character of employment transitions. Unfortunately, the window of observation in our study was too short to study this properly. A secondary comparison of characteristics across employment transitions over time showed that people who remained unemployed became slightly higher educated over time. Other characteristics and health outcomes, however, remained stable over time (results not shown), suggesting that changing characters of the transitions over time is unlikely to explain our findings.

Our study has a number of strengths. The longitudinal availability of work status data, as well as other variables such as income, allowed us to study single transitions in employment status and persistence in unemployment over multiple years. Our study also benefits from the use of linkage with register data to objectively ascertain the work status. A limitation, however, is that persons not included in our analyses were generally less healthy than included participants, which means we cannot exclude some selection bias. Furthermore, selection bias may be present because we were not able to distinguish unemployed persons who gave up their job search from those still trying to get a job. It is plausible that discouraged non-workers have lower career chances and worse health, which may have led us to underestimate any association between single transitions and persistence in unemployment and health. Another limitation is that health data was measured only once. We therefore do not know if persons were in worse health before their health was measured and if their work status changed because of it. Indeed, findings from a secondary analysis in our study showed that individuals with better health were more likely to be (persistently) employed and vice versa (results not shown). Hence, replication of our study using registered longitudinal health data would strengthen our findings and may also provide insight in the development of health outcomes over time. Studies taking the main reason for entry into unemployment into account would further disentangle the existence of possible selection effects [16]. Unfortunately, based on our data no further differentiation with respect to this aspect was possible. Furthermore, although the study population was rather large, the decreasing number of persons with increased persistence of unemployment resulted in estimates with larger confidence intervals for these groups. In addition, although we adjusted for a range of confounders, residual confounding due to unmeasured factors, such as ethnicity and financial strain cannot be excluded. Finally, it has earlier been suggested that the health status of employees may be negatively affected by job insecurity [10, 51–53]. Unfortunately, we did not have data available on types of contracts (i.e. temporarily or permanent) and whether job loss was involuntary or not. It is likely that voluntary job loss has attenuated the observed findings toward the null, leading to an underestimate of the estimates. Research focusing on precariousness of the new employment and health status would provide valuable information concerning this topic.

## Conclusions

Summarising, our observational findings suggest that single transitions and persistence in unemployment are associated with higher likelihood of poor mental and general health, obesity, and to a lesser extent smoking. These associations seemed to be partly, but not fully attributable to a range of confounders and personal income. In line with previous studies, this study implies that re-employment might be an important strategy to improve health of unemployed individuals. This holds true, in particular, for individuals who have been unemployed for a longer duration in time. The relatively extensive Dutch social security system may explain our findings that the economic recession did not modify the observed associations between transitions in employment status and poorer health. Nevertheless, findings from other prospective studies with longitudinal measures of health status are needed to confirm our findings.

## Data Availability

The microdata are not publically available, but are available on request from Statistics Netherlands, by sending a completed application form (see https://www.cbs.nl/en-gb/our-services/customised-services-microdata/microdata-conducting-your-own-research/applying-for-access-to-microdata) to aanvraagmicrodata@cbs.nl.

## Appendix 1

**Table 5:** Comparison of baseline characteristics of included versus excluded individuals in analyses on single transitions in employment status and general health

Characteristic	Included *N* = 47,734	Excluded^a^ *N* = 10,170	*p*-value
Age, y (mean ± SD)	41.6 ± 12.2	46.8 ± 11.5	< 0.001
Men (%, N)	52.4 (25014)	52.1 (5303)	0.64
Intermediate education (%,N)	38.7 (18475)	44.4 (1897)	< 0.001
Lower education (%,N)	9.1 (4329)	5.5 (234)	< 0.001
BMI ≥ 30 k/m^2^ (%,N)	10.2 (4887)	11.8 (1198)	< 0.001
Poor perceived health (%,N)	15.2 (7248)	17.5 (1776)	< 0.001
Poor mental health (%,N)	11.3 (3723)	12.0 (895)	0.11
Current smokers (%,N)	30.1 (14385)	25.9 (2630)	< 0.001

^a^Individuals excluded due to missing data

## Appendix 2

**Table 6:** Odds Ratios and 95% confidence intervals for the associations between single transitions in employment status and persistence of unemployment and poor general health, obesity and smoking^a^

	Poor general health	Obesity	Smoking
Single transitions in employment status^b^			
Employed-employed	1.00 (Reference)	1.00 (Reference)	1.00 (Reference)
Unemployed-employed	1.56 (1.25,1.95)*	1.32 (1.02,1.71)*	1.37 (1.14,1.65)*
Employed-unemployed	1.89 (1.57,2.26)*	1.08 (0.85,1.38)	1.75 (1.49,2.05)*
Unemployed-unemployed	4.67 (4.24,5.14)*	1.54 (1.36,1.73)*	1.44 (1.31,1.58)*
P-trend	< 0.001	< 0.001	< 0.001
Persistence of unemployment in the past 5 years^cd^			
0 (=persistently employed)	1.00 (Reference)	1.00 (Reference)	1.00 (Reference)
1	1.16 (0.94,1.42)	1.33 (1.06,1.66)*	1.78 (1.51,2.09)*
2	1.56 (1.20,2.02)*	1.48 (1.09,1.99)*	1.47 (1.17,1.84)*
3	1.82 (1.32,2.51)*	1.34 (0.90,2.01)	1.53 (1.14,2.06)*
4	2.92 (2.01,4.23)*	1.47 (0.91,2.39)	1.76 (1.24,2.52)*
5 (=persistently unemployed)	5.13 (3.85,6.84)*	2.13 (1.53,2.98)*	1.20 (0.93,1.54)
P-trend	< 0.001	< 0.001	0.25

^a^Adjusted sex, age, education, unemployment in the region, household position and personal gross income. ^b^ Single transitions in employment status relate to the work transitions in the previous 2 years. ^c^ Persistence in unemployment relates to the number of years individuals were unemployed in the previous 5 years. ^d^ To account for the last registered work status in the analysis of persistence of unemployment, we additionally adjusted for individual’s employment status (i.e. employed or unemployed) at the time of the health survey

**p* ≤ 0.05

## Abbreviations

BMI: Body Mass Index;
CI: Confidence Interval
GPD: Gross domestic product
HIS: Health Interview Survey
MHI-5: Mental Health Inventory (MHI-5)
OR: Odds Ratio
